# Theory of Weakly Polydisperse Cytoskeleton Filaments

**DOI:** 10.3390/polym14102042

**Published:** 2022-05-17

**Authors:** Vadim Warshavsky, Marcelo Marucho

**Affiliations:** Department of Physics and Astronomy, The University of Texas at San Antonio, San Antonio, TX 78249, USA; vadim_varshavskiy@yahoo.com

**Keywords:** cytoskeleton filaments, second virial coefficient, phase behavior, polyelectrolytes

## Abstract

Cytoskeleton filaments have the extraordinary ability to change conformations dynamically in response to alterations of the number density of actins/tubulin, the number density and type of binding agents, and the electrolyte concentration. This property is crucial for eukaryotic cells to achieve specific biological functions in different cellular compartments. Conventional approaches to biopolymers’ solution break down for cytoskeleton filaments because they entail several approximations to treat their polyelectrolyte and mechanical properties. In this article, we introduce a novel density functional theory for polydisperse, semiflexible cytoskeleton filaments. The approach accounts for the equilibrium polymerization kinetics, length and orientation filament distributions, as well as the electrostatic interaction between filaments and the electrolyte. This is essential for cytoskeleton polymerization in different cell compartments generating filaments of different lengths, sometimes long enough to become semiflexible. We characterized the thermodynamics properties of actin filaments in electrolyte aqueous solutions. We calculated the free energy, pressure, chemical potential, and second virial coefficient for each filament conformation. We also calculated the phase diagram of actin filaments’ solution and compared with the corresponding results in in vitro experiments.

## 1. Introduction

Eukaryotic cells can dynamically regulate the biological environment and the polyelectrolyte and mechanical properties of cytoskeleton filaments to achieve specific biological functions as diverse as directional growth, shape, division, plasticity, and migration [1]. For instance, an increase in the number density of G-actin/tubulin and electrolyte concentration can lead to conformation transformations from the orientation-disordered (isotropic) to orientation-ordered (nematic) phase, as well as increasing the filaments’ average length. Additionally, a growth in the number density of binding agents, such as divalent ions or linker proteins, can yield bundling or network conformations [2]. These self-organization behaviors, yet poorly understood, have been observed experimentally. Currently, valuable information on the distribution and type of cytoskeleton conformations in cells is obtained from fluorescence and electron microscopy images [3,4,5,6], whereas confocal microscopy captures their dynamic conformation changes [7,8,9]. However, this information usually provides an incomplete molecular understanding of the interplay between the polydispersity, semiflexibility, polyelectrolyte, and mechanical properties of cytoskeleton filaments on their conformational dynamics, self-organization, and stability. This understanding is crucial to elucidate the biophysical principles underlying fundamental biological functions of eukaryotic cells in normal and pathological conditions, which may vary depending on the cell type and location, gender, age, and inheritance conditions.

A substantial amount of theoretical research has been performed in the field to study the isotropic to nematic phase transformation in macromolecules’ solution. The conventional understanding of the properties of these polyelectrolytes is based on monodisperse (e.g., same filament lengths), mean-field theories, and rod-like cylindrical filament models (e.g., with contour lengths shorter than their persistence length). These methods break down for cytoskeleton filaments because they entail several approximations to treat the inter-filament interactions, electrolytes, and filament structures. Onsager [10] showed that the expansion of the free energy functional up to the second virial coefficient provides accurate results for monodisperse long charged rods in the coexisting isotropic and nematic phases. In the nematic phase, the free energy is expressed as a functional of angular distribution functions of rods, which minimizes the free energy functional. Onsager introduced a trial function for the angular distribution functions depending on a single parameter to increase the efficiency of these calculations. Subsequently, a variety of modifications and extensions of Onsager’s theory were proposed [11,12]. For instance, Odijk introduced a Gaussian-type trial function for the angular distribution. While less accurate than Onsager’s approximation, it overcame some limitations on the numerical calculations. It provides explicit, analytic expressions for the thermodynamic properties that indeed facilitated the phase diagram transition and coexistence analysis [13]. A different approach was proposed to estimate the angular distribution functions for monodisperse, long cytoskeleton filament rods [2]. They were not obtained by minimizing the free energy, but postulating a particular form in each phase with a single variational parameter characterizing the width of the angular distributions.

Additionally, corrections were made to the orientational part of Onsager’s free energy to consider monodisperse macromolecules having contour lengths larger than or the same order as their persistence length [14,15]. Furthermore, later approximations were developed to patch the orientational free energies for rigid and semiflexible macromolecules [13,16]. A different approach was introduced by Sluckin [17], who generalized Onsager’s theory to describe polydisperse rigid rods having the same Gaussian form for the size distribution function in both isotropic and nematic phases. Moreover, Odijk’s ansatz was generalized to the polydisperse case, where the nematic phase onset was formed by rods with lengths larger than the average length of the size distribution in the isotropic phase. Different modifications of the method were proposed to address this shortcoming [18,19,20,21]. In particular, the input size asymmetric distribution functions in the form of Schultz’s and log-normal distributions were considered.

On the other hand, a particular approach was introduced for amphiphilic micellar suspensions [22]. The size distribution function of micellar macromolecules was not considered as an input value, but rather the standard chemical potential that governs the size–angular distribution functions [23]. It was found that the micella average lengths in the coexisting isotropic and nematic phases are different, with larger micella sizes in the nematic phase.

The size distribution function of uncharged polydisperse actin filament rods in the bundling phase was calculated from a free energy that accounts for hard-core filament repulsion and short-range attractions [24]. It was shown that short-range attractions, arising either from linker proteins, depletion-mediated attractions, or polyvalent ions, enhance the tendency of filaments to align parallel to each other, yielding an increase in the average filament length and a decrease in the relative width of the distribution of filament lengths.

These approaches for phase diagram studies consider specific macromolecular properties, neglecting others. For instance, some approaches focused on polyelectrolyte and polydispersity properties only, whereas other approximations accounted for semiflexibility and polyelectrolyte properties, and so on. However, for cytoskeleton filaments, it is imperative to consider the balance and competition between contributions coming from their polydispersity, semiflexibility, polyelectrolyte, and mechanical properties to the total free energy. When accounting for all of these features, one can formulate a more accurate and realistic description of the conformational dynamics, self-organization, and stability properties of cytoskeleton filaments in different cell compartments.

As a first step to face this challenge, we introduce in this article a novel density functional theory for polydisperse, semiflexible cytoskeleton filaments. The approach accounts for the equilibrium polymerization kinetics, length and orientation filament distributions, as well as the electrostatic interaction between filaments and the electrolyte. As a unique feature, the formulation is able to determine critical parameter values governing the isotropic–nematic phase diagram behavior. This approach is essential to study the self-organization behavior of actin filaments taking place in different cell compartments, where the G-actin polymerization and electrolyte conditions may generate filaments with different conformations and lengths long enough to become semiflexible. Specifically, we consider experimental conditions on the actin filaments’ solution where the isotropic–nematic phase diagram transition is due to changes in the G-actin concentration. Additionally, the filament average size for different G-actin concentrations was fixed by changing the gelsolin proteins’ concentration [25,26]. From these special equilibrium conditions, we obtained the size distribution function, whereas we used Sluckin’s trial function to calculate the angular distribution function in the nematic phase. Additionally, we introduced an ansatz for the standard chemical potential excess of actin filaments that results in the asymmetric Schulz distribution function for the actin filaments size. This distribution function agrees with those used in light-scattering experiments on actin filaments [25]. In addition, we generalized the formula for the orientational free energy introduced in [13] to the case of a polydisperse system to account for the filament semiflexibility. Finally, we calculated the isotropic–nematic phase diagrams for a variety of Schulz size distributions, persistence lengths, and concentrations of monovalent ions in the electrolyte solution. We also compared these results with available experimental data [26].

The paper is organized as follows. The theory is described in Section 2; the numerical results are given in Section 3; the discussion is provided in Section 4; the details of the calculations are presented in Appendix A, Appendix B, Appendix C, Appendix D and Appendix E.

## 2. Materials and Methods

We considered a solution of polydisperse actin filaments, each of them having a length $L=l_{m}\nu$, where $l_{m}$ is the length of an actin monomer unit and ν the number of monomer units representing the filament size. Each filament has its own direction in 3D space, which is characterized by a body-angle ω accounting for the chosen nematic director. In spherical coordinates, we have dω=sinθdθdφ with the *z* direction taken along the nematic axis.

The size–angular density distribution function of actin filaments $\rho_{\nu }\left(\omega \right)$ is given by the following expression:

(1) $$\begin{array}{c} \rho_{\nu }\left(\omega \right)=\rho n_{\nu }\eta_{\nu }\left(\omega \right), \end{array}$$

where ρ=N/V is the total density of the filaments, *N* is the total filament number of any size, and *V* represents the volume of the system. Basically, $\rho_{\nu }\left(\omega \right)$ represents the number of filaments of size ν oriented along the direction ω. Additionally, $n_{\nu }$ is the size distribution function averaged over all angles, and $\eta_{\nu }\left(\omega \right)$ represents the angular distribution functions, which also depend on the filament size ν. The normalization conditions for the size- and angular-distribution functions $n_{\nu }$ and $\eta_{\nu }\left(\omega \right)$ are

(2) $$\begin{array}{c} \sum_{\nu \geq 1}n_{\nu }=1, \end{array}$$

and

(3) $$\begin{array}{c} \int \eta_{\nu }\left(\omega \right)d\omega =1, \end{array}$$

respectively. The summation in Equation (2) is performed over all filament sizes ν, whereas the integration in Equation (3) is carried out over the whole body angle. The size distribution function $n_{\nu }$ is characterized by the filament average size <ν>, namely the average degree of polymerization, as well as the normalized standard deviation σ, such as

(4) $$\begin{array}{c} <\nu >=\sum_{\nu \geq 1}\nu n_{\nu }, \end{array}$$

and

(5) $$\begin{array}{c} \sigma^{2}=\frac{<\nu^{2}>-<\nu {>}^{2}}{<\nu {>}^{2}}, \end{array}$$

where

(6) $$\begin{array}{c} <\nu^{2}>=\sum_{\nu \geq 1}\nu^{2}n_{\nu }. \end{array}$$

### 2.1. The Free Energy Density Functional

We define the dimensionless Helmholtz free energy of cytoskeleton filaments *f* as follows:

(7) $$\begin{array}{c} f=\frac{\beta F}{N}=f_{ig}+f_{int}+\sum_{\nu \geq 1}\int \beta \mu_{\nu }^{\left(0\right)}\rho_{\nu }\left(\omega \right)d\omega , \end{array}$$

where *F* is the free energy, $\beta =1/k_{B}T$ the inverse thermal energy, $k_{B}$ the Boltzmann constant, *T* the temperature, $f_{ig}$ the dimensionless ideal gas free energy, $f_{int}$ the dimensionless energy due to the inter-filament interactions, and $\mu_{\nu }^{\left(0\right)}$ the standard chemical potential of ν-sized filaments.

The expressions for $f_{ig}$ and $f_{int}$ as functionals of the density distributions $n_{\nu }$ and $\eta_{\nu }\left(\omega \right)$ are provided below.

#### 2.1.1. Ideal Gas Free Energy $f_{ig}$

The ideal gas free energy per volume of the system *V* can be written in the following form:

(8) $$\begin{array}{c} \frac{\beta F_{ig}}{V}=\sum_{\nu \geq 1}\int \rho_{\nu }\left(\omega \right)\left(\ln[4\pi \rho_{\nu }\left(\omega \right)\Lambda_{\nu }^{3}]-1\right)d\omega , \end{array}$$

where $\Lambda_{\nu }=\frac{h}{\sqrt{2\pi m_{\nu }k_{B}T}}$ is the thermal de Broglie wavelength of ν-sized filaments, *h* is the Planck constant, and $m_{\nu }=\nu m_{1}$ stands for the ν-sized filament mass. Substitution of Equation (1) into Equation (8) yields

(9) $$\begin{array}{c} f_{ig}=\left(\ln\rho \Lambda_{1}^{3}-1\right)\sum_{\nu \geq 1}n_{\nu }\int \eta_{\nu }\left(\omega \right)d\omega +\sum_{\nu \geq 1}n_{\nu }\ln n_{\nu }\int \eta_{\nu }\left(\omega \right)d\omega \\ -\sum_{\nu \geq 1}n_{\nu }s_{\nu }^{\left(or\right)}+\int \rho_{\nu }\left(\omega \right)\ln\left(\nu^{-\frac{1}{3}}\right)d\omega , \end{array}$$

where $f_{ig}=\beta F_{ig}/N$ is the ideal gas free energy per filament (N=ρV), $\Lambda_{1}=\frac{h}{\sqrt{2\pi m_{1}k_{B}T}}$ the monomer thermal de Broglie wavelength, and $s_{\nu }^{\left(or\right)}$ the filament orientational entropy:

(10) $$\begin{array}{c} s_{\nu }^{\left(or\right)}=-\int \eta_{\nu }\left(\omega \right)\ln\left[4\pi \eta_{\nu }\left(\omega \right)\right]d\omega . \end{array}$$

#### 2.1.2. Interaction Free Energy $f_{int}$

We write the interaction free energy per volume *V* in a mean-field fashion, namely

(11) $$\begin{array}{c} \frac{\beta F_{int}}{V}=\frac{1}{2}\sum_{\nu_{1}\geq 1}\sum_{\nu_{2}\geq 1}\int \rho_{\nu_{1}}\left(\omega_{1}\right)\rho_{\nu_{2}}\left(\omega_{2}\right)B_{\nu_{1}\nu_{2}}\left(\omega_{1},\omega_{2}\right)d\omega_{1}d\omega_{2}. \end{array}$$

where $B_{\nu_{1}\nu_{2}}\left(\omega_{1},\omega_{2}\right)$ represents the cluster integral

(12) $$\begin{array}{c} B_{\nu_{1}\nu_{2}}\left(\omega_{1},\omega_{2}\right)=\int d{\vec{r}}_{12}\left[1-e^{-\beta w_{12}\left(r_{12},\omega_{1},\omega_{2}\right)}\right], \end{array}$$

In Equation (12), $w_{12}$ is the interaction potential between two filaments and $r_{12}$ the closest distance between them. The expression (11) is a good approximation for the interaction free energy of monodisperse rigid charged rods. In fact, it becomes exact in the limit of infinitely long rods, i.e., for L/D→∞ [10].

Substitution of Equation (1) into Equation (11) yields the following expression for $F_{int}$ as a functional of the densities $n_{\nu }$ and $\eta_{\nu }\left(\omega \right)$:

(13) $$\begin{array}{c} \frac{\beta F_{int}}{V}=\frac{\rho^{2}}{2}\sum_{\nu_{1}\geq 1}\sum_{\nu_{2}\geq 1}\;n_{\nu_{1}}n_{\nu_{2}}\int \eta_{\nu_{1}}\left(\omega_{1}\right)\eta_{\nu_{2}}\left(\omega_{2}\right)B_{\nu_{1}\nu_{2}}\left(\omega_{1},\omega_{2}\right)d\omega_{1}d\omega_{2}. \end{array}$$

To calculate the cluster integral $B_{\nu_{1}\nu_{2}}\left(\omega_{1},\omega_{2}\right)$ in obvious form, we model the interaction potential between two charged rod filaments $w_{12}\left(r_{12},\omega_{1},\omega_{2}\right)$ as follows:

(14) $$\begin{array}{c} \beta w_{12}\left(r_{12},\omega_{1},\omega_{2}\right)=\left\{\begin{array}{cc} +\infty ,\;\;\;\;\;\;\;\;\;\;\;\; & r_{12}<D\\ \Gamma (r_{12},\gamma_{12}),\;\;\;\;\;\;\;\;\;\;\;\; & D\leq r_{12} \end{array}\right. \end{array}$$

where *D* is the bare rod diameter and $\gamma_{12}$ the angle between them. The function $\Gamma (r_{12},\gamma_{12})$ represents the following repulsive electrostatic inter-filament interaction potential: [27]

(15) $$\Gamma \left(r_{12},\gamma_{12}\right)=\frac{\Gamma_{⊥}}{\sin\gamma_{12}}e^{-k_{D}\left(r_{12}-D\right)},$$

where

(16) $$\Gamma_{⊥}=\frac{2\pi \lambda^{2}\beta }{\varepsilon k_{D}}\frac{e^{-k_{D}D}}{{\left[\frac{k_{D}D}{2}K_{1}\left(\frac{k_{D}D}{2}\right)\right]}^{2}}.$$

In Equation (16), $k_{D}={\left(4\pi \beta e^{2}/\varepsilon \sum_{i=1}^{2}\xi_{i}\rho_{i}\right)}^{\frac{1}{2}}$ stands for the inverse Debye length, $K_{1}$ the modified Bessel function of second kind of first order, λ the filament linear charge density, ε the water solvent dielectric permittivity, *e* the electron charge, and $\xi_{i}$ and $\rho_{i}$ the ion valency and concentration of species *i* in solution, respectively. We note that Equation (15) comes from the solution of the linearized Poisson–Boltzmann equation, which is accurate for monovalent ions, namely $\left|\xi_{i}\right|=1$. Substitution of Equations (14) and (15) into Equation (12) results in the following expression for the cluster integral $B_{\nu_{1},\nu_{2}}\left(\omega_{1},\omega_{2}\right)$

(17) $$\begin{array}{c} B_{\nu_{1},\nu_{2}}\left(\omega_{1},\omega_{2}\right)=2D_{eff}l_{m}^{2}\nu_{1}\nu_{2}B*\left(\sin\gamma_{12}\right), \end{array}$$

where $D_{eff}$ and $B*\left(\sin\gamma_{12}\right)$ are the effective diameter and the dimensionless function given by Equations (A12) and (A14), respectively. The details of these calculations are presented in Appendix A.

Furthermore, substitution of Equation (17) into Equation (13) provides the following form for the interaction free energy:

(18) $$\begin{array}{c} f_{int}=\rho B_{2}, \end{array}$$

where

$$B_{2}\equiv \frac{\pi }{4}D_{eff}l_{m}^{2}\sum_{\nu_{1}\geq 1}\sum_{\nu_{2}\geq 1}\;n_{\nu_{1}}n_{\nu_{2}}\nu_{1}\nu_{2}h_{\nu_{1}\nu_{2}}$$

is the extension of the second virial coefficient for polydisperse filaments, and the function $h_{\nu_{1}\nu_{2}}$ is given by

(19) $$\begin{array}{c} h_{\nu_{1}\nu_{2}}=\frac{4}{\pi }\int \eta_{\nu_{1}}\left(\omega_{1}\right)\eta_{\nu_{2}}\left(\omega_{2}\right)B*\left(\sin\gamma_{12}\right)d\omega_{1}d\omega_{2}. \end{array}$$

We note that the parameter $h_{\nu_{1}\nu_{2}}$ accounts for the orientational averaged contributions coming from the excluded volume and charge density interactions between filaments of size $\nu_{1}$ and $\nu_{2}$, as well as the influence of the ionic strength on the second virial coefficient.

It is worth mentioning that the solution of the linearized Poisson–Boltzmann equation in Equations (14)–(16) is valid for infinitely long filaments. Indeed, some correction terms should be considered for short filaments to account for the end effects. However, we considered filaments of average size units of monomers 450÷5000, which correspond to filament lengths in the range of 1.2÷13.5 μm only. Thus, the linearized Poisson–Boltzmann equation solution is justified in our study.

### 2.2. The Distribution Functions

We introduce the Lagrange functional:

(20) $$\begin{array}{c} f^{\prime }=f+A\left(\sum_{\nu \geq 1}n_{\nu }-1\right)+\sum_{\nu \geq 1}n_{\nu }B_{\nu }\left(\int \eta_{\nu }\left(\omega \right)d\omega -1\right) \end{array}$$

to find the distribution functions, where *f* represents the free energy functional given by Equation (7), whereas *A* and $B_{\nu }$ are the Lagrange multipliers accounting for the normalization conditions given by Equations (2) and (3), respectively. For a fixed angular distribution function $\eta_{\nu }\left(\omega \right)$, the equilibrium size distribution function $n_{\nu }$ minimizes the Lagrange functional. Thus, we have

(21) $$\begin{array}{c} \frac{\partial f^{\prime }}{\partial n_{\nu }}=0. \end{array}$$

Using the chain rule, the l.h.s. of Equation (21) can be rewritten in the following form:

(22) $$\begin{array}{c} \frac{\partial f^{\prime }}{\partial n_{\nu }}=\sum_{\lambda \geq 1}\mu_{\lambda }^{\prime }\;\left(\frac{\partial n_{\lambda }}{\partial n_{\nu }}\right), \end{array}$$

where

(23) $$\begin{array}{c} \mu_{\lambda }^{\prime }=\frac{\partial f^{\prime }}{\partial n_{\lambda }}. \end{array}$$

An additional relationship for the distribution functions $\left\{n_{\nu }\right\}$ comes from Equation (4). Differentiation on both sides of Equation (4) yields

(24) $$\begin{array}{c} dn_{1}+\sum_{\nu \geq 2}\nu dn_{\nu }=0. \end{array}$$

As a result, Equation (22) can be written as follows:

(25) $$\begin{array}{c} \frac{\partial f^{\prime }}{\partial n_{\nu }}=\mu_{1}^{\prime }\;\frac{\partial n_{1}}{\partial n_{\nu }}+\sum_{\lambda \geq 2}\mu_{\lambda }^{\prime }\;\left(\frac{\partial n_{\lambda }}{\partial n_{\nu }}\right). \end{array}$$

Substitution of Equation (24) into the r.h.s. of Equation (25) leads to

(26) $$\begin{array}{c} \frac{\partial f^{\prime }}{\partial n_{\nu }}=-\mu_{1}^{\prime }\nu +\mu_{\nu }^{\prime }. \end{array}$$

Consequently, Equations (21) and (26) yield

(27) $$\begin{array}{c} \mu_{\nu }^{\prime }=\mu_{1}^{\prime }\nu . \end{array}$$

It is worth noting that ν-sized filaments can be considered as separated “pseudo-phases”. Additionally, the “pseudo-phases” with all possible sizes ν are in equilibrium with each other if the equilibrium condition given by Equation (27) is executed. In fact, the value for $\mu_{\nu }^{\prime }$ in Equation (27) does not represent the real chemical potential of ν-sized filaments since it comes from the Lagrange functional $f^{\prime }$ in Equation (23), rather than the Helmholtz free energy *f*. To calculate $\mu_{\nu }^{\prime }$, we substitute Equation (20) into Equation (23) and use Equations (3), (7), (9), and (18). We obtain

(28) $$\begin{array}{c} \beta \mu_{\nu }^{\prime }=\ln n_{\nu }-\ln C+\frac{\pi }{2}Dl_{m}^{2}\rho \;\nu \sum_{\nu^{\prime }\geq 1}n_{\nu^{\prime }}\nu^{\prime }h_{\nu \nu^{\prime }}+\beta \mu_{\nu }^{\left(0\right)}, \end{array}$$

where *C* is the following constant:

(29) $$\begin{array}{c} -\ln C=\ln\rho \Lambda_{1}^{3}-s_{\nu }^{\left(or\right)}+A. \end{array}$$

We substitute the expression (28) into the condition of equilibrium (27) to obtain the following equation for the length distribution function $n_{\nu }$:

(30) $$\begin{array}{c} \ln\frac{n_{\nu }}{C}=\nu \ln\frac{n_{1}}{C}-\frac{\pi }{2}Dl_{m}^{2}\rho \;\nu \sum_{\nu^{\prime }\geq 1}n_{\nu^{\prime }}\nu^{\prime }\left(h_{\nu \nu^{\prime }}-h_{1\nu^{\prime }}\right)-\beta \Delta \mu_{\nu }^{\left(0\right)}, \end{array}$$

where $\Delta \mu_{\nu }^{\left(0\right)}$ denotes the standard chemical potential difference between ν actin units aggregated in a ν-mer filament $\mu_{\nu }^{\left(0\right)}$ and the one in the single dispersed phase $\nu \mu_{1}^{\left(0\right)}$, i.e.,

(31) $$\begin{array}{c} \Delta \mu_{\nu }^{\left(0\right)}=\mu_{\nu }^{\left(0\right)}-\nu \mu_{1}^{\left(0\right)}. \end{array}$$

Finally, we substitute the parameter $y\equiv -\ln(\frac{n_{1}}{C})$ into Equation (30) to obtain the following master equation for $n_{\nu }$:

(32) $$\begin{array}{c} n_{\nu }=Ce^{-\nu y}e^{-\beta \Delta \mu_{\nu }^{\left(0\right)}}e^{-\frac{\pi }{2}Dl_{m}^{2}\rho \;\nu \sum_{\nu^{\prime }\geq 1}n_{\nu^{\prime }}\nu^{\prime }\left(h_{\nu \nu^{\prime }}-h_{1\nu^{\prime }}\right)}. \end{array}$$

Similarly, for a fixed distribution function $n_{\nu },$ the angular distribution function $\eta_{\nu }\left(\omega \right)$ is obtained by using the variational principle:

(33) $$\begin{array}{c} \frac{\partial f^{\prime }}{\partial \eta_{\nu }\left(\omega \right)}=0. \end{array}$$

Substitution of Equation (20) into Equation (33) and the use of Equations (2), (4), (7), (9), (10), (18), and (19) yield

(34) $$\eta_{\nu }\left(\omega_{1}\right)=E_{\nu }e^{-2Dl_{m}^{2}\;\rho \nu \;\sum_{\nu^{\prime }\geq 1}n_{\nu^{\prime }}\nu^{\prime }\int \eta_{\nu^{\prime }}\left(\omega_{2}\right)\sin\gamma_{12}d\omega_{2}},$$

where $E_{\nu }$ is a constant that satisfies the following relationship:

(35) $$\begin{array}{c} -\ln E_{\nu }=\ln\rho \Lambda^{3}+\ln n_{\nu }+\beta \mu_{\nu }^{\left(0\right)}+B_{\nu }. \end{array}$$

To obtain the expression for $E_{\nu }$, we integrate both sides of Equation (34) with respect to $\omega_{1}$ and use the normalization conditions (2) and (3). Finally, we replace the expression obtained for $E_{\nu }$ into Equation (34) to obtain the following master equation for $\eta_{\nu }\left(\omega \right)$:

(36) $$\eta_{\nu }\left(\omega_{1}\right)=\frac{e^{-2D_{eff}l_{m}^{2}\;\rho \nu \;\sum_{\nu^{\prime }\geq 1}n_{\nu^{\prime }}\nu^{\prime }\int \eta_{\nu^{\prime }}\left(\omega_{2}\right)\sin\gamma_{12}d\omega_{2}}}{\int d\omega_{1}e^{-2D_{eff}l_{m}^{2}\;\rho \nu \;\sum_{\nu^{\prime }\geq 1}n_{\nu^{\prime }}\nu^{\prime }\int \eta_{\nu^{\prime }}\left(\omega_{2}\right)\sin\gamma_{12}d\omega_{2}}}.$$

Furthermore, the angular distribution function $\eta_{\nu }\left(\omega \right)$ depends only on the polar angle θ due to the symmetry of the system. Thus, we have $\eta_{\nu }\left(\omega \right)=\eta_{\nu }\left(\theta \right)$.

To find the size and angular distribution functions $n_{\nu }$ and $\eta_{\nu }\left(\theta \right)$, we solve the expressions (32) and (36) using the successive iterations method. To this end, we chose the monodisperse size distribution as the initial guess for the first iteration step, i.e., $n_{\nu }=\delta \left(\nu -<\nu >\right)$, where δ is a Dirac delta function. Substitution of this expression for $n_{\nu }$ into Equation (36) generates the following equation to numerically calculate η(θ) [28]:

(37) $$\eta \left(\theta_{1}\right)=\frac{e^{-2D_{eff}l_{m}^{2}\;\rho \;<\nu {>}^{2}\int \eta \left(\theta_{2}\right)\sin\gamma_{12}d\omega_{2}}}{\int d\omega_{1}e^{-2D_{eff}l_{m}^{2}\;\rho \;<\nu {>}^{2}\int \eta \left(\theta_{2}\right)\sin\gamma_{12}d\omega_{2}}}.$$

More efficient, but less accurate values for the monodisperse angular distribution function η(θ) were proposed using some functional forms. For instance, Onsager [10] introduced the trial function η(θ)=αcosh(αcosθ)/(4πsinhα). Another commonly used, although slightly less-accurate ansatz is the so-called Odijk’s trial function [13]:

(38) $$\eta \left(\theta \right)=\frac{\alpha }{4\pi }e^{-\frac{\alpha }{2}\theta^{2}}.$$

We note that these trial functions depend on a single unknown parameter α, which is chosen to minimize the free energy functional *f*.

In the second iterative step, we substitute the angular distribution function η(θ) obtained in the first step into Equations (19) and (32)to calculate $n_{\nu }$. Since η(θ) does not depend on ν, it follows from Equation (19) that $h_{\nu \nu^{\prime }}-h_{1\nu^{\prime }}=0$. Additionally, we use in Equation (32) the ansatz:

(39) $$\begin{array}{c} \beta \Delta \mu_{\nu }^{\left(0\right)}=-z\ln\nu \end{array}$$

to obtain a size distribution function $n_{\nu }$ in the asymmetric Schulz–Zimm form [29]:

(40) $$\begin{array}{c} n_{\nu }=\frac{y^{z+1}}{\Gamma (z+1)}e^{-\nu y}\nu^{z}, \end{array}$$

where *z* represents the conventional polydispersity parameter, whereas $C=\frac{y^{z+1}}{\Gamma (z+1)}$ is the constant coming from the normalization condition given by Equation (2). In Appendix B, we show that the polydispersity parameter *z* is related to the normalized standard deviation of the size distribution function $\sigma =\frac{1}{\sqrt{z+1}}$, whereas the parameter *y* depends on the values <ν> and *z* only, i.e., y=(z+1)/<ν>.

Finally, substitution of Equation (40) into Equation (36) gives an equation to calculate the function $\eta_{\nu }\left(\theta \right)$. Obtaining the numerical solution of this equation indeed requires a high computational cost because it depends on two variables ν and θ. Alternatively, we generalize the monodisperse Odijk’s trial function given by Equation (38) to the polydisperse case. Specifically, we introduce the following parametrization for the polydisperse angular distribution function [17]:

(41) $$\eta_{\nu }\left(\theta \right)=\frac{\alpha_{\nu }}{4\pi }e^{-\frac{\alpha_{\nu }}{2}\theta^{2}},$$

where the polydisperse Gaussian parameter $\alpha_{\nu }$ depends on the filament size ν. For the weak polydispersity of the system, the ratio $\frac{\Delta \nu }{<\nu >}\equiv \frac{\nu -<\nu >}{<\nu >}$ can be considered a small parameter. As a result, the polydisperse Gaussian parameter $\alpha_{\nu }$ can be calculated using the following linear expansion around the monodisperse solution:

(42) $$\alpha_{\nu }=\alpha \;\left(1+\gamma \frac{\Delta \nu }{<\nu >}\right),$$

where the two unknown parameters α and γ are chosen to minimize the free energy functional *f*. In the monodisperse limit, we have that Δν→0 and $\alpha_{\nu }\to \alpha$.

### 2.3. Free Energy in the Nematic Phase

We substitute the normalization conditions (2) and (3) into Equations (7) and (9) to write the Helmholtz free energy per filament as follows:

(43) $$\begin{array}{c} f=\ln\rho \Lambda_{1}^{3}-1+f_{or}+f_{int}+f_{mix}+\sum_{\nu \geq 1}\beta \mu_{\nu }^{\left(0\right)}n_{\nu }, \end{array}$$

where $\mu_{\nu }^{\left(0\right)}\to \mu_{\nu }^{\left(0\right)}+\nu^{1/3}$ and the terms $f_{or}$, $f_{int}$, $f_{mix}$ represent the orientational, interaction, and mixing free energies, respectively. The expressions for these energies are provided below.

#### 2.3.1. Orientational Free Energy

The orientational free energy in Equation (43) is given by the expression:

(44) $$\begin{array}{c} f_{or}=\sum_{\nu \geq 1}n_{\nu }\int \eta_{\nu }\left(\theta \right)\ln\left[4\pi \eta_{\nu }\left(\theta \right)\right]d\omega . \end{array}$$

Substitution of the expressions for $\eta_{\nu }\left(\theta \right)$ (41) and (42) into Equation (44) yields

(45) $$f_{or}=\ln\alpha -1-\frac{\gamma^{2}}{2}\sigma^{2}.$$

Equation (45) represents the generalization of the orientational free energy expression obtained by Odijk for monodisperse rods, i.e., for filaments with contour length <L>≪P, where *P* is the filament persistence length. Indeed, in the monodisperse limit σ→0, the correct limit $f_{or}\to \ln\alpha -1$ is obtained for any value of the parameter γ [13]. For flexible filaments, where <L>≫P, the orientational free energy can be written in the following form [13]:

(46) $$f_{or}=\sum_{\nu \geq 1}n_{\nu }\frac{L}{8P}\int \frac{1}{\eta_{\nu }\left(\theta \right)}{\left(\frac{\partial \eta_{\nu }\left(\theta \right)}{\partial \theta }\right)}^{2}d\omega .$$

Substitution of the expressions for $\eta_{\nu }\left(\theta \right)$ (41) and (42) into Equation (46) leads to

(47) $$f_{or}=\frac{N_{p}\alpha }{4},$$

where

(48) $$N_{p}\equiv \frac{<L>}{P}\left(1+\gamma \sigma^{2}\right)$$

and $<L>=l_{m}<\nu >$. Certainly, in the monodisperse limit, σ→0 the correct limit $f_{or}\to \frac{L}{P}\frac{\alpha }{4}$ is obtained [11].

To calculate the orientational free energy for any persistence length, we combine these two asymptotic cases for $f_{or}$ using the following interpolating formula:

(49) $$f_{or}=\ln\alpha -\frac{\gamma^{2}}{2}\sigma^{2}+N_{p}\frac{\left(\alpha -1\right)}{6}+\frac{5}{12}\ln\left[\cosh\left(N_{p}\frac{\left(\alpha -1\right)}{5}\right)\right]-\frac{19}{12}\ln2.$$

The details on these calculations are provided in Appendix C.

#### 2.3.2. Interaction Free Energy

We substitute Equations (40)–(42) into Equations (18) and (19) to obtain

(50) $$\begin{array}{c} f_{int}=\rho \;B_{2}^{N}, \end{array}$$

where

(51) $$\begin{array}{c} B_{2}^{N}\equiv \frac{\pi }{4}D_{eff}l_{m}^{2}<\nu {>}^{2}h_{z} \end{array}$$

represents the second virial coefficient for polydisperse filaments in the nematic phase and

(52) $$\begin{array}{c} h_{z}=32\;\int_{0}^{\pi /2}\sin\theta_{1}d\theta_{1}\int_{0}^{\pi /2}\sin\theta_{2}d\theta_{2}g_{z}\left(\theta_{1}\right)g_{z}\left(\theta_{2}\right)K\left(\theta_{1},\theta_{2}\right), \end{array}$$

(53) $$\begin{array}{c} g_{z}\left(\theta \right)=\frac{\alpha }{4\pi }e^{-\frac{\alpha }{2}\left(1-\gamma \right)\theta^{2}}\left(\frac{\gamma \frac{\left(z+2\right)}{\left(z+1\right)}}{{\left(1+\frac{\gamma \alpha \theta^{2}}{2(z+1)}\right)}^{z+3}}+\frac{\left(1-\gamma \right)}{{\left(1+\frac{\gamma \alpha \theta^{2}}{2(z+1)}\right)}^{z+2}}\right), \end{array}$$

and

(54) $$\begin{array}{c} K\left(\theta_{1},\theta_{2}\right)=\int_{0}^{2\pi }B*\left(\sin\gamma_{12}\right)d\varphi_{12}. \end{array}$$

The details on these calculations are presented in Appendix D.

Equation (50) represents the generalization of the interaction free energy expression for polydisperse actin filaments. In fact, in the monodisperse limit z→∞, the expression for $g_{z}\left(\theta \right)$ goes to

(55) $$\begin{array}{c} g_{z}\left(\theta \right)\to \eta \left(\theta \right)\;\;\;\;\;\;\;\;\;\;\;\;\;\;\left(z\to \infty \right), \end{array}$$

which is the monodisperse distribution function given by Equation (38). Similarly, Equations (50) and (52)–(54) recover the correct expression for the filaments’ interaction free energy $f_{int}$ in the monodisperse case:

(56) $$\begin{array}{c} f_{int}=\rho \;\frac{\pi }{4}D_{eff}L^{2}h, \end{array}$$

with

(57) $$\begin{array}{c} h=32\;\int_{0}^{\pi /2}\sin\theta_{1}d\theta_{1}\int_{0}^{\pi /2}\sin\theta_{2}d\theta_{2}\eta \left(\theta_{1}\right)\eta \left(\theta_{2}\right)K\left(\theta_{1},\theta_{2}\right). \end{array}$$

#### 2.3.3. Mixing Free Energy

The mixing free energy in Equation (43) can be written as:

(58) $$\begin{array}{c} f_{mix}=\sum_{\nu \geq 1}n_{\nu }\ln n_{\nu } \end{array}.$$

This expression is not well defined in the monodisperse limit (z→∞) since the density distribution $n_{\nu }\to \delta \left(\nu -<\nu >\right)$ and the mixing free energy diverges, rather than going to zero. To overcome this shortcoming, we extract the density ρ-independent term $f_{mix}\left(z\to \infty \right)$ in Equation (A45) from the r.h.s. of Equation (58). The resulting renormalized mixing free energy reads

(59) $$\begin{array}{c} f_{mix}=\sum_{\nu \geq 1}n_{\nu }\ln n_{\nu }+\ln\left[\sqrt{2\pi e}\;<\nu >\sigma \right]. \end{array}$$

This new expression (59) recovers the correct value in the monodisperse limit, i.e., $f_{mix}\to 0$ for σ→0 (z→∞). More details on these calculations are provided in Appendix E.

### 2.4. Free Energy in the Isotropic Phase

In the isotropic phase, there is no preferential direction for the filament orientations; thus, the orientational distribution function $\eta_{\nu }\left(\theta \right)=1/\left(4\pi \right)$ for any filament length, and the orientational free energy becomes

(60) $$\begin{array}{c} f_{or}=0. \end{array}$$

The expression for the interaction free energy is obtained from Equations (4), (18), and (19). We have

(61) $$\begin{array}{c} f_{int}=\rho B_{2}^{I}, \end{array}$$

where $B_{2}^{I}$ is the second virial coefficient for polydisperse filaments in the isotropic phase

(62) $$\begin{array}{c} B_{2}^{I}=\frac{\pi }{4}D_{eff}l_{m}^{2}<\nu {>}^{2}h, \end{array}$$

with

(63) $$h=\frac{2}{\pi }\int_{0}^{\pi }B*\left(\gamma_{12}\right)\sin\gamma_{12}d\gamma_{12}.$$

Finally, substitution of Equation (A14) into Equation (63) generates the following expression for *h* in the isotropic phase

(64) $$\begin{array}{c} h=1+\frac{1}{k_{D}D_{eff}}\;\frac{2}{\pi }\int_{0}^{\pi }d\gamma_{12}\;\;\sin^{2}\gamma_{12}\;E_{1}\left(\frac{\Gamma_{⊥}}{\sin\gamma_{12}}\right), \end{array}$$

where $E_{1}\left(x\right)$ is the elliptic integral given by Equation (A9).

### 2.5. Isotropic–Nematic Phase at Equilibrium

The isotropic–nematic phase equilibrium in a system of polydisperse macromolecules occurs when the chemical potentials of each species $\mu_{\nu }$ and the osmotic pressures Π are equal to each other. Specifically, we have [17,19,20]

(65) $$\begin{array}{cc} \mu_{\nu }^{\left(I\right)}=\mu_{\nu }^{\left(N\right)}\;\;\;\;\;\;\;\;\;\;\;\; & (\nu \geq 1), \end{array}$$

(66) $$\begin{array}{c} \Pi^{\left(I\right)}=\Pi^{\left(N\right)}. \end{array}$$

In the case of the self-aggregation of actin filaments, there is an additional condition for the chemical equilibrium in each thermodynamic phase, namely

(67) $$\begin{array}{c} \mu_{\nu }=\nu \mu_{1}, \end{array}$$

where $\mu_{\nu }=\frac{\partial F}{\partial N_{\nu }}$ is the chemical potential of ν-sized filaments and $\mu_{1}$ the chemical potential of actin monomers. The condition (67) is obtained using a method similar to the one leading to Equation (27). Substitution of Equation (67) into Equation (65) provides a single equilibrium condition for the chemical potentials, i.e.,

(68) $$\begin{array}{c} \mu_{1}^{\left(I\right)}=\mu_{1}^{\left(N\right)}. \end{array}$$

To calculate the chemical potential of actin monomers $\mu_{1}$, we write the Gibbs free energy of mixture of actin filaments of all sizes in the following form:

(69) $$\begin{array}{c} \frac{G}{N}=\sum_{\nu \geq 1}\mu_{\nu }n_{\nu }. \end{array}$$

Substitution of Equations (4) and (67) and the use of condition $\frac{G}{N}=\frac{F}{N}+\frac{\Pi }{\rho }$ in Equation (69) yield

(70) $$\mu_{1}=\frac{1}{<\nu >}\left(\frac{F}{N}+\frac{\Pi }{\rho }\right).$$

In Equations (66) and (70), we use the formula $\Pi =\rho^{2}\frac{\partial }{\partial \rho }\left(\frac{F}{N}\right)$ for practical calculations of the osmotic pressure.

Using Equation (4), we can also write the average filaments’ length in Equation (70) as follows:

(71) $$\begin{array}{c} <\nu >=\frac{\rho_{A}}{\rho }, \end{array}$$

where $\rho_{A}=\sum_{\nu \geq 1}\nu \rho n_{\nu }$ is the G-actin number density. As a result, the average filaments length represents the ratio of *G*-actin and *F*-actin concentrations. Additionally, *G*-actin is usually polymerized in in vitro experiments in the presence of gelsolin, an actin-binding protein known to cap and sever actin filaments [25,26,30]. It was noticed in these experiments that the concentration of F-actin in the solution becomes almost equal to the concentration of gelsolin, i.e., $\rho \approx \rho_{G}$. As a result, the average length of filaments (average degree of polymerization) <ν> in these experiments was regulated by the gelsolin concentration. In [26], the variation of the concentration of *G*-actin $\rho_{A}$ was accompanied by a change in the concentration of gelsolin $\rho_{G}$ to keep the average length of filaments <ν> fixed during the isotropic–nematic phase transition. Using this experimental condition in our theory, the mixing free energy $f_{mix}$ and the term with $\mu_{\nu }^{\left(0\right)}$ in the r.h.s. of the expression for free energy (43) are also fixed values and, thus, do not contribute to the phase coexistence properties.

Thus, using Equations (43), (50), and (61), the dimensionless free energy *f* in the nematic and isotropic phases can be written as follows:

(72) $$\begin{array}{c} f\left(c\right)=\ln c-1+f_{or}+c\;H, \end{array}$$

where *c* represents the dimensionless filaments’ density:

(73) $$\begin{array}{c} c=\rho \frac{\pi }{4}D_{eff}l_{m}^{2}<\nu {>}^{2}. \end{array}$$

The expressions to calculate $f_{or}$ are given by Equations (49) and (60) for the nematic and isotropic phases, respectively, whereas the corresponding expressions for *H* are given by Equations (52) and (64).

In the nematic phase, the free energy *f* (72) is a function of the parameters of the angular distribution function α, γ, and the dimensionless filaments’ density *c*; thus, f≡f(c;α,γ). By minimizing *f*, we obtain the equilibrium parameter functions $\alpha_{eq}\left(c\right)$ and $\gamma_{eq}\left(c\right)$ for each value of *c*. Thus, the expression for the nematic free energy at equilibrium becomes $f_{eq}\left(c\right)=f\left(c,\alpha_{eq}\left(c\right),\gamma_{eq}\left(c\right)\right)$. The minimization is carried out using the downhill simplex method in multiple dimensions [31]. Finally, the coexisting dimensionless densities $c_{I}$ and $c_{N}$ are obtained from the coexistence conditions (66) and (68), whereas the corresponding actin densities $\rho_{A}^{I}$ and $\rho_{A}^{N}$ are obtained from Equations (71) and (73).

In principle, the chemical potential of the non-depleted unimers (G-actin) $\mu_{1}$ and the depleted actin concentration $\rho_{A}$ are related by Equation (70). We calculate the Helmholtz free energy in Equation (72) as a function of $\rho_{A}$ in the isotropic or nematic phase. Subsequently, we substitute the result into the r.h.s. of Equation (70) to obtain the relationship $\mu_{1}=\mu_{1}\left(\rho_{A}\right)$ or, vice versa, $\rho_{A}=\rho_{A}\left(\mu_{1}\right)$. In the last case, the chemical potential of unimers $\mu_{1}$ can be regarded as an input parameter, which is set by the reservoir condition.

## 3. Results

In this section, we apply the approach to investigate the isotropic–nematic phase diagram for polydisperse actin filaments in monovalent salt solutions. We considered typical experimental values for key parameters, such as the filament persistence length, the average filament size, the polydispersity parameter, and the electrolyte concentration to elucidate their impact on the orientational and size distributions. We also calculated the thermodynamic properties of the system, including the free energy, pressure, chemical potential and, consequently, the range of G-actin concentrations and filaments’ average lengths leading to conformation transformations from the orientation-disordered (isotropic) to orientation-ordered (nematic) phase. In the numerical calculations, we chose the values for the filaments’ diameter $D=80\;\overset{˚}{A}$, the monomer units’ length $l_{m}=27\;\overset{˚}{A}$, the actin molar weight $m_{A}=42\;$kDa, and the linear filament charge density $\lambda =-4\frac{e}{\mathrm{nm}}$.

We calculated the isotropic and nematic coexisting dimensionless densities for rigid monodisperse rods with length L=1 μm for the electrolyte’s concentration $c_{e}=0.1\;$M to compare our results with those obtained by Borukhov et al. [2]. Our result was $c_{I}=3.82$ and $c_{N}=5.10$, whereas the corresponding one in [2] was $c_{I}=4.25$ and $c_{N}=9.98$ (see Figure 3 in [2]). The source of such discrepancy is associated with the cone approximation used in [2] for the angular distribution function.

### 3.1. Distribution Functions

**Figure 1:**
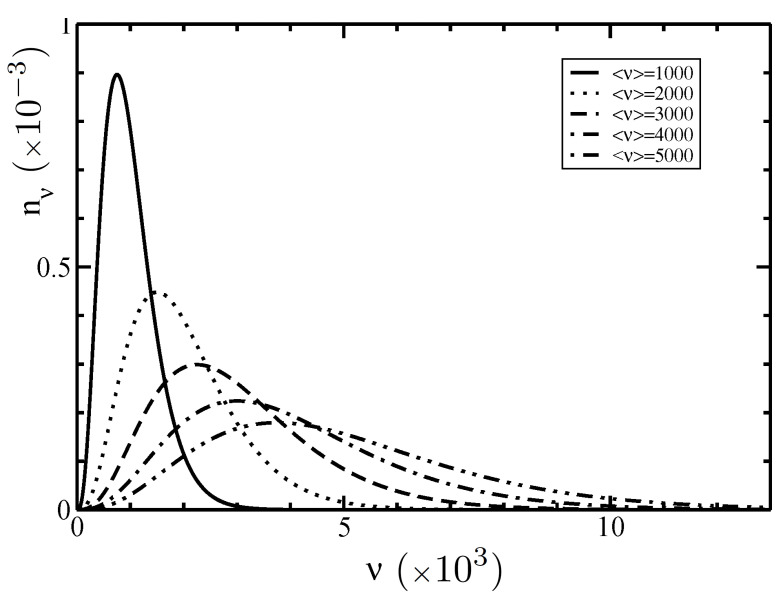
Schulz length distribution function $n_{\nu }$ as a function of the filament size ν for different average sizes <ν> from Equation (40). The normalized standard deviation is σ=0.5 (z=3).

In this section, we present the analysis on the distribution functions $n_{\nu }$ and $\eta_{\nu }\left(\theta \right)$ in the I–N phase coexistence for polydisperse, semiflexible filaments. We used a persistence length P=18 μm and an electrolyte concentration $c_{e}=0.1\;$M. In Figure 1, we plot the Schulz length distribution function $n_{\nu }$ for different average sizes <ν> in a weakly polydisperse system with normalized standard deviation σ=0.5 (z=3).

Our results revealed polydisperse distribution functions with lower peaks and broadened distributions for larger values of the average size <ν>, while the asymmetric behavior characterizes the different increasing rate lengths of barbed and pointed ends. This asymmetry property plays a crucial role in the phase diagram behavior. Since the normalization integral $\int_{1}^{\infty }n_{\nu }d\nu =1$ can be split into the sum of two integrals $\int_{1}^{<\nu >}n_{\nu }d\nu =0.57$ and $\int_{<\nu >}^{\infty }n_{\nu }d\nu =0.43$ (z=3), each of them is independent of the value <ν>, and the amount of the filaments with the sizes ν shorter than <ν> is larger than the one with the sizes longer than <ν>. In contrast, monodisperse, symmetric distribution functions are represented by delta function distributions $n_{\nu }\to \delta \left(\nu -<\nu >\right)$.

**Figure 2:**
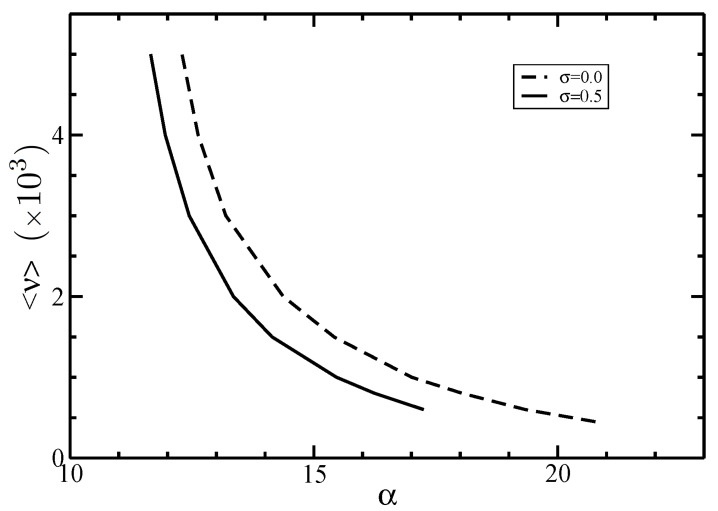
The parameter α as a function of the average size <ν> at the I–N phase coexistence. The normalized standard deviations are σ=0 (dashed line) and 0.5 (solid line).

**Figure 3:**
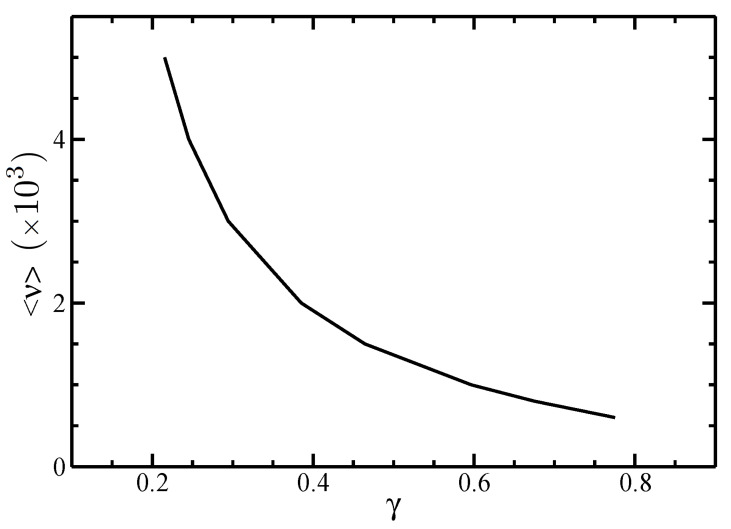
The parameter γ as a function of the average size <ν> at the I–N phase equilibrium. The normalized standard deviation is σ=0.5.

In Figure 2 and Figure 3, we depicted the I–N phase coexistence values of the parameters α and γ appearing in the angular distribution function for different values of the average length <ν>.

It is seen that a larger average length <ν> leads to lower parameter values α and γ. In fact, larger filament sizes generate narrower angular distributions, increasing the order of the system. For a given value of <ν>, Figure 2 also shows that the parameter α decreases with increasing the polydispersity parameter σ. This is due to the typical broadened size distributions that characterize polydisperse systems, which include short filaments, lowering the order of the system.

**Figure 4:**
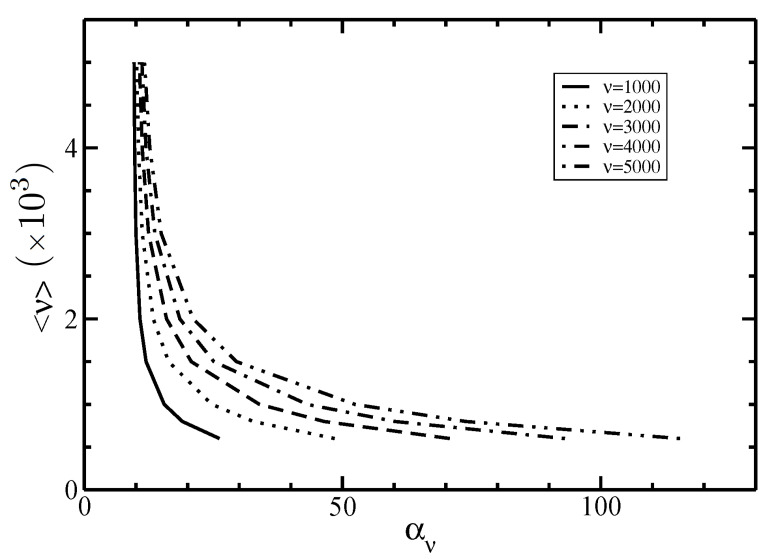
The Gaussian parameter $\alpha_{\nu }$ vs. the average size <ν> at I–N phase coexistence for different lengths of filaments ν.

**Figure 5:**
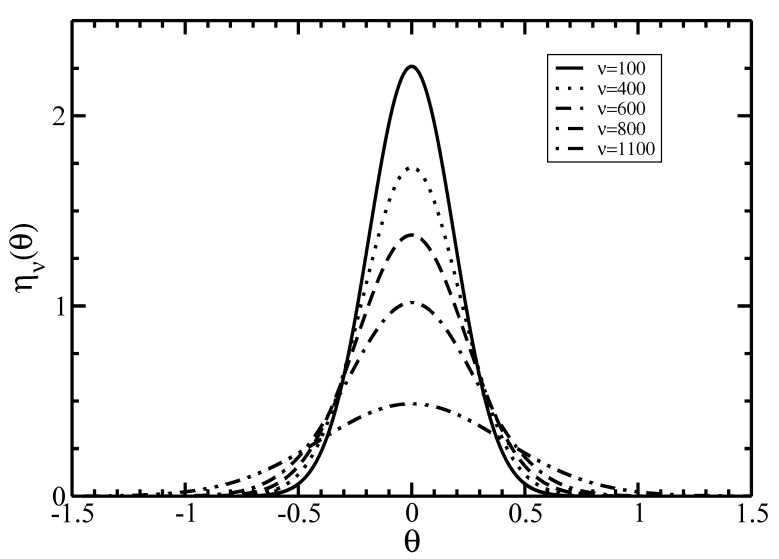
The angular distribution function $\eta_{\nu }\left(\theta \right)$ as a function of the angle θ at the I–N phase coexistence for different sizes ν. The average length is <ν>=600.

In Figure 4, we plot the Gaussian parameter $\alpha_{\nu }$ given in Equation (42) as a function of the average filament size ν. For a fixed size ν, the parameter $\alpha_{\nu }$ decreases with increasing the average length <ν>. Additionally, the dependence of the orientational distribution function $\eta_{\nu }\left(\theta \right)$ on the angle θ for different sizes ν is plotted in Figure 5.

**Figure 6:**
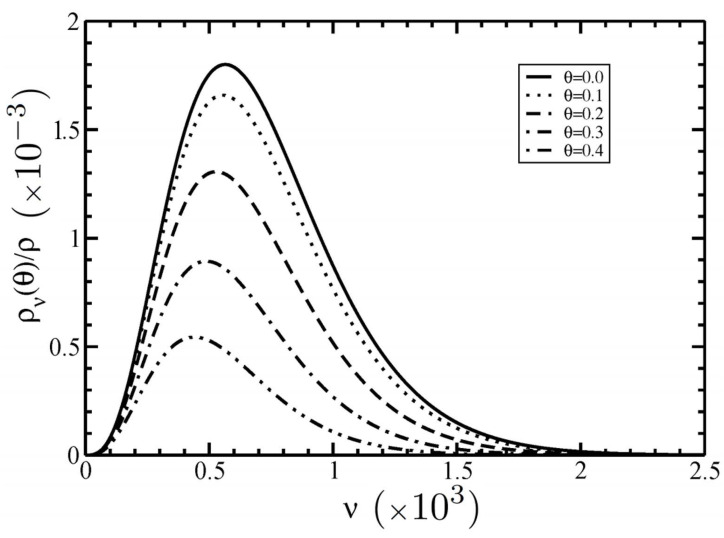
The size–angular distribution function $\rho_{\nu }\left(\theta \right)/\rho =n_{\nu }\eta_{\nu }\left(\theta \right)$ as a function of the size ν at the I–N phase coexistence for different angles θ. The average length is <ν>=600.

It is seen that the increase of size ν flattens and widens the distribution $\eta_{\nu }\left(\theta \right)$. On the other hand, the dependence of the total size–angular distribution function $\rho_{\nu }\left(\theta \right)/\rho =n_{\nu }\eta_{\nu }\left(\theta \right)$ on the filament size ν for several angles θ is displayed in Figure 6.

Our results showed that the increase of the angle θ flattens the total distribution $\rho_{\nu }\left(\theta \right)/\rho$. This is because most filaments in the nematic phase are oriented along the nematic director with θ=0. By increasing the angle θ, the amount of filaments decreases and is directed along the direction with angle θ.

### 3.2. Isotropic–Nematic Phase Diagram

**Figure 7:**
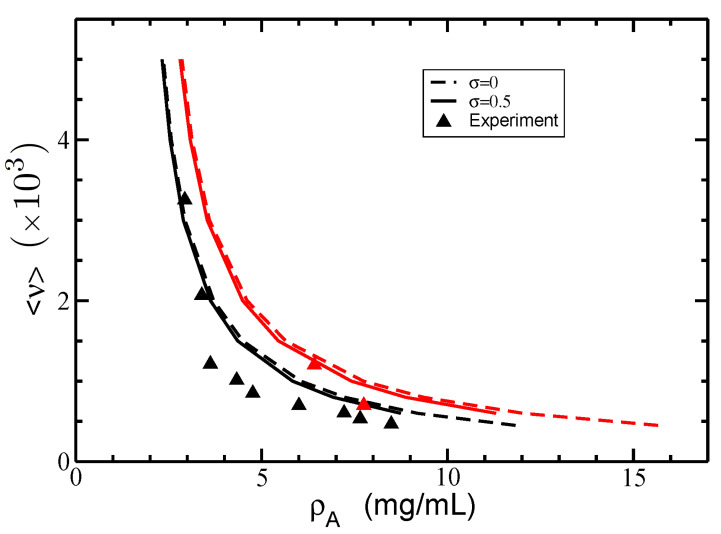
The isotropic–nematic phase diagram in terms of coexisting densities of actin $\rho_{A}$ for different average lengths of filaments <ν> and two values of the normalized standard deviations σ=0 (dashed lines) and σ=0.5 (solid lines). The persistence length is P=18 μm, and the electrolyte concentration $c_{e}=0.1\;$M. The experimental data (triangles) were retrieved from [26]. The coexisting isotropic and nematic densities are plotted in black and red colors, respectively.

In Figure 7, we plot the I–N phase diagram of actin filaments in terms of the coexisting dimensional densities of actin $\rho_{A}$ for different average lengths <ν> and two values for the normalized standard deviation of Schulz distribution function: σ=0 (monodisperse) and σ=0.5 (weakly polydisperse). In these calculations, we used the values P=18 μm and $c_{e}=0.1\;$M. In the same figure, we plot the experimental data extracted from Figures 4 and 5 in [26].

Our results showed isotropic and nematic metastable phases represented by the area enclosed between the red and black curves, whereas the area to the left of the black curves and to the right of the red curves defines the exclusive randomly oriented (isotropic) and parallel oriented (nematic) phases, respectively. Additionally, the red and black curves display an increase in the average filament length with decreasing G-actin concentration and, consequently, the order of the system. For a given G-actin concentration, the nematic phase generates larger average filament lengths as compared to the isotropic phase, whereas higher G-actin concentrations are required in the nematic phase to generate the same average filament length obtained in the isotropic phase. Figure 7 also shows a minor dependence of the coexisting densities on the polydispersity parameter. This is in part due to the experimental condition fixing the same degree of polymerization <ν> for both the isotropic and nematic phases. In fact, Figure 7 shows that polydispersity with a larger amount of short filaments slightly reduces both isotropic and nematic coexisting densities as compared to the monodisperse case. Thus, additional factors such as electrostatic effects compete with hard-body interactions to reduce the coexisting densities in the polydisperse case.

**Figure 8:**
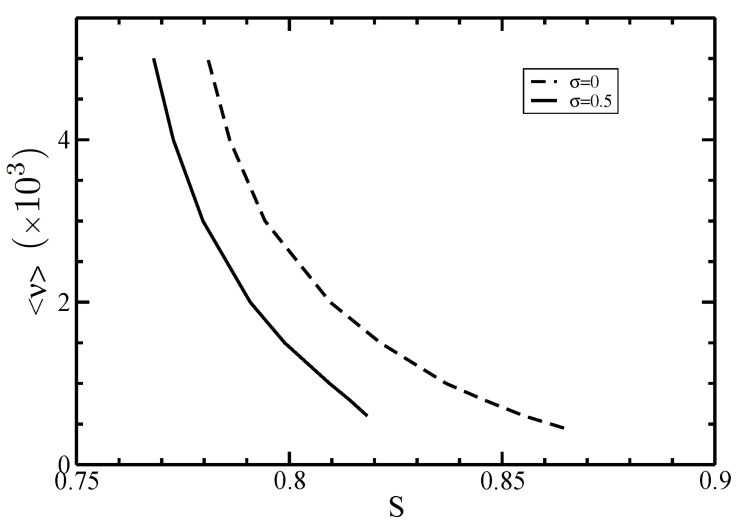
The nematic order parameter *S* at the I–N phase coexistence for different average lengths <ν>. The normalized standard deviation are σ=0 (dashed line) and 0.5 (solid line).

We also studied the polydispersity effects on the nematic (anisotropic) order parameter *S*, which is defined as follows:

(74) $$S=\sum_{\nu \geq 1}n_{\nu }\int d\omega \eta_{\nu }\left(\theta \right)P_{2}\left(\cos\theta \right),$$

where $P_{2}=\left(3\cos^{2}\theta -1\right)/2$ is the Legendre polynomial of second order. In the isotropic phase, where $\eta_{\nu }\left(\theta \right)$ is constant, the order parameter *S* vanishes. In contrast, in a system highly aligned, $\eta_{\nu }\left(\theta \right)$ becomes the Dirac delta function leading to S=1. The I–N phase coexistence values of the nematic order parameter *S* for different average sizes <ν> and two values of the polydispersity parameter σ are displayed in Figure 8. For both values of σ, our results revealed a decrease of the order parameter *S* with increasing the average length <ν>. Moreover, the order parameter *S* is even smaller for polydisperse filaments.

Overall, we realized that the orientational order of filaments at I–N phase equilibrium is due to the competition between two contributions. In the previous section, we showed that the increase of the average length <ν> leads to narrower angular distributions and, thus, higher order in the filaments orientation. On the other hand, Figure 7 displays a decrease in the coexisting nematic density, lowering their order. Since the order parameter *S* accounts for both contributions and the total order becomes smaller for larger average lengths <ν>, we conclude that the contribution coming from the decrease in the coexisting nematic density dominates the order behavior of the filaments over those arising from narrowed angular distributions.

**Figure 9:**
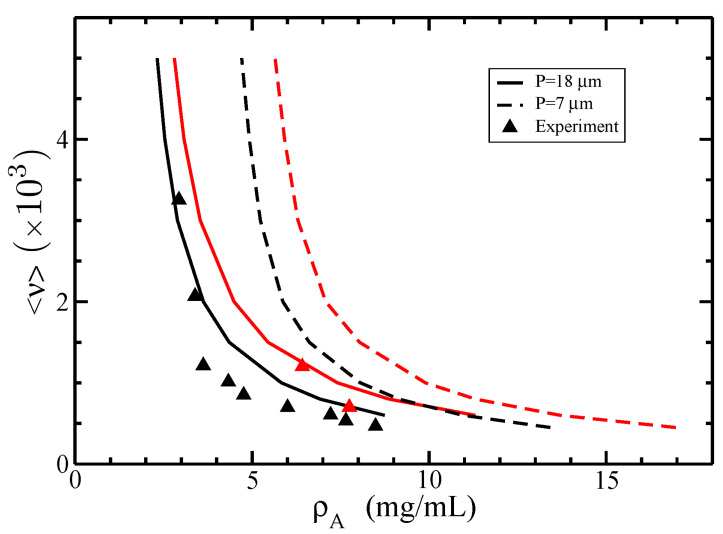
The isotropic–nematic phase diagram in terms of coexisting densities of actin $\rho_{A}$ for different average lengths of filaments <ν> and two values of persistence lengths P=7 μm (dashed lines) and P=18 μm (solid lines). The normalized standard deviation is σ=0.5, and the electrolyte concentration $c_{e}=0.1\;$M. The experimental data (triangles) were retrieved from [26]. The coexisting isotropic and nematic densities are plotted in black and red colors, respectively.

We also considered several filament semiflexibility parameter values since they vary depending on the polymerization buffers, experimental protocols, and techniques. We analyze in Figure 9 the isotropic–nematic phase diagram for two typical values of the persistence length, namely P=7 μm and P=18 μm, in physiological conditions (electrolyte concentration $c_{e}=0.1\;$M).

For a given value <ν>, we found that larger values of the persistence length *P* decrease both the isotropic and nematic coexisting densities. Additionally, the results for P=18 μm give the best fit against the experimental data. This result stems from the impact of the semiflexibility on the orientational free energy $f_{or}$. It can be seen from Equation (49) that the increase of the persistence length *P* leads to the decrease of the orientational term $f_{or}$, which boosts the formation of the nematic state and generates smaller values for the coexisting densities.

**Figure 10:**
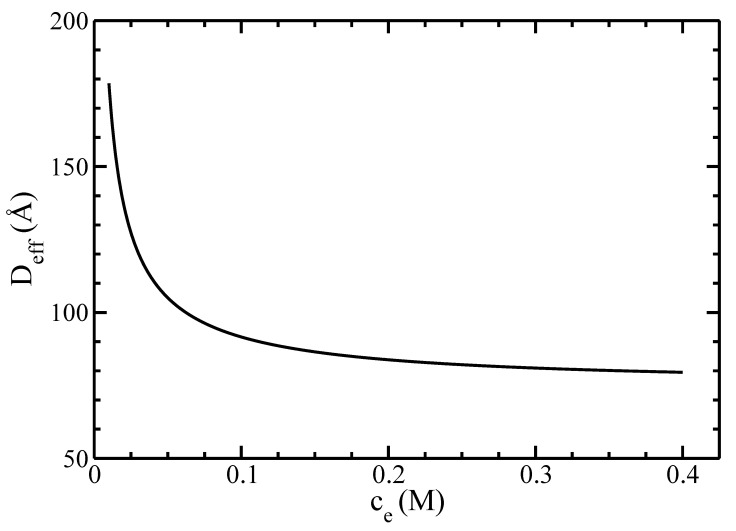
The effective diameter $D_{eff}$ as a function of the monovalent ions’ concentration $c_{e}$.

Finally, we studied the effects of the electrolyte’s concentration on the I–N phase diagram of actin filaments. We plot in Figure 10 the dependence of the effective diameter $D_{eff}$ on the concentration of monovalent ions $c_{e}$.

Our results show an exponential decay of $D_{eff}$ with increasing $c_{e}$. This result agrees with the formation of an electrical double-layer accumulating many counterions around the filament surface. The lower the electrolyte concentration, the larger its thickness and, consequently, the effective filament diameter are. For salt concentrations larger than 0.4M, the effective diameter of filaments $D_{eff}$ is smaller than the diameter of filaments $D=80\;\overset{˚}{A}$, and the calculations may become inaccurate.

**Figure 11:**
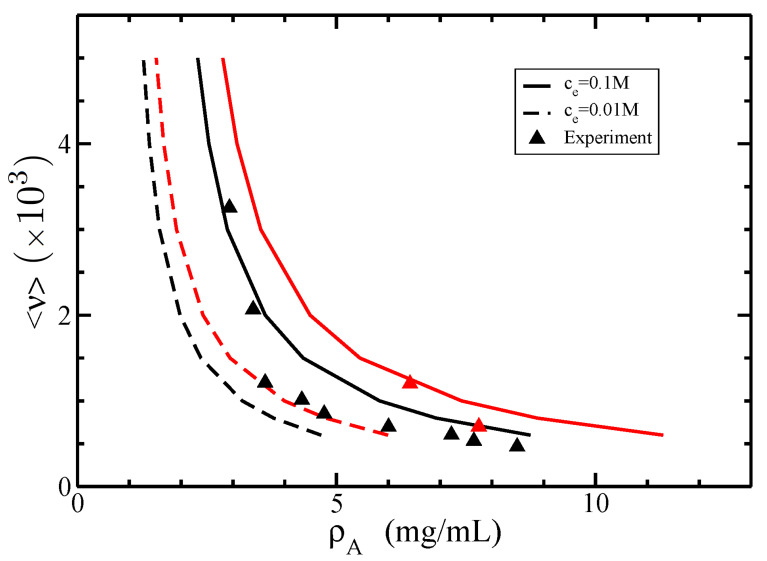
The isotropic–nematic phase diagram in terms of coexisting densities of actin $\rho_{A}$ for different average lengths of filaments <ν> and two values of electrolyte concentrations $c_{e}=0.01\;$M (dashed lines) and $c_{e}=0.1\;$M (solid lines). The persistence length is P=18 μm, and the normalized standard deviation σ=0.5. The experimental data (triangles) were retrieved from [26]. The coexisting isotropic and nematic densities are plotted in black and red colors, respectively.

Furthermore, we show in Figure 11 the isotropic–nematic phase diagram for two values of electrolyte concentration $c_{e}=0.01\;$M and $c_{e}=0.1\;$M. It is seen that the increase in electrolyte concentration leads to the increase in both coexisting isotropic and nematic densities.

Clearly, the results for $c_{e}=0.1\;$M give the best fit against the experimental data. This behavior is due to a substantial decrease of the effective diameter $D_{eff}$ with increasing the electrolyte concentration $c_{e}$ (see Figure 10), as well as because the coexisting density $\rho_{A}$ is inversely proportional to the effective diameter (see Equation (73)).

**Figure 12:**
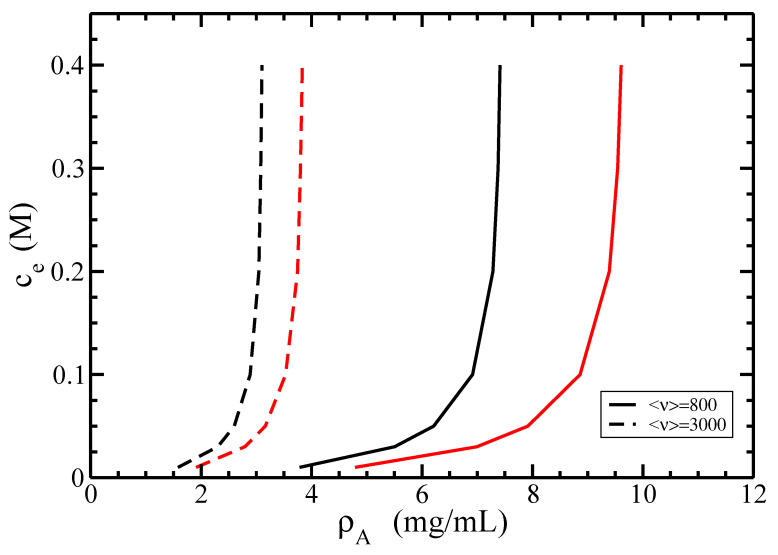
The isotropic–nematic phase diagram in terms of coexisting densities of actin $\rho_{A}$ for different values of the monovalent ions’ concentrations $c_{e}$. The average lengths of filaments are <ν> = 800 (solid lines) and 3000 (dashed lines); the persistence length P=18 μm, and the normalized standard deviation σ=0.5. The coexisting isotropic and nematic densities are plotted in black and red colors, respectively.

In Figure 12, we investigate the I–N phase diagram for a fixed average length <ν> in terms of the dependence of the coexisting densities of actin $\rho_{A}$ on the concentration of monovalent ions $c_{e}$.

It is seen that the increase of the concentration $c_{e}$ leads to the increase of both isotropic and nematic coexisting densities $\rho_{A}$. On the other hand, for a fixed value of $c_{e}$, the increase of the average size <ν> decreases both coexisting densities $\rho_{A}$. Certainly, it is easier for long rods or filaments to form a nematic order rather than for shorter ones. Similarly, for longer filaments (<ν>=3000), the I–N phase transition occurs at lower densities $\rho_{A}$ compared to short ones (<ν>=800) with the same concentration $c_{e}$.

**Figure 13:**
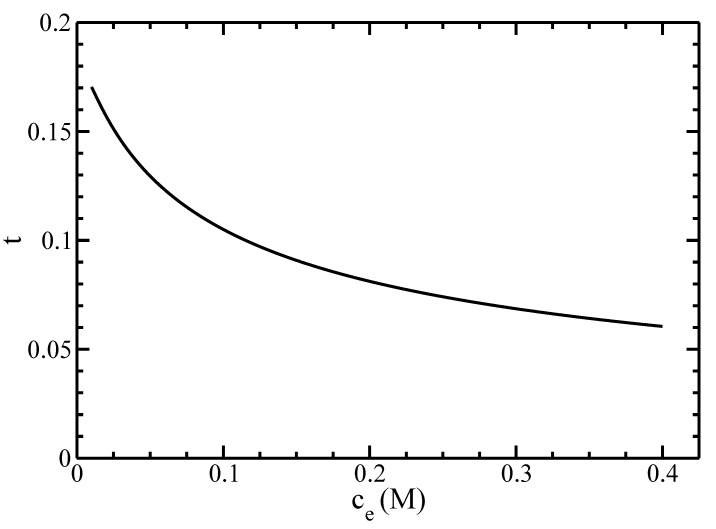
The twisting parameter *t* as a function of the monovalent ions’ concentration $c_{e}$.

Another quantity playing a key role in the I–N phase diagram is the twisting parameter. In some studies [12], the terms with exponential integral $E_{1}\left(t\right)$ in the formulas analogous to Equations (A14) and (64) were dropped for simplicity, such as the electrostatic effects on the phase equilibrium can be described exclusively by the effective diameter $D_{eff}$ and the twisting parameter:

(75) $$t=\frac{1}{k_{D}D_{eff}},$$

which is a measure of a twist of equally charged cylinders, which hinders the formation of the nematic order. On another hand, the increase of the effective diameter $D_{eff}$ promotes the nematic order. The competition between these two effects affects the I–N phase equilibrium in the electrolyte solution [12]. We plot the dependence of the twisting parameter *t* on the concentration of monovalent ions $c_{e}$ in Figure 13.

**Figure 14:**
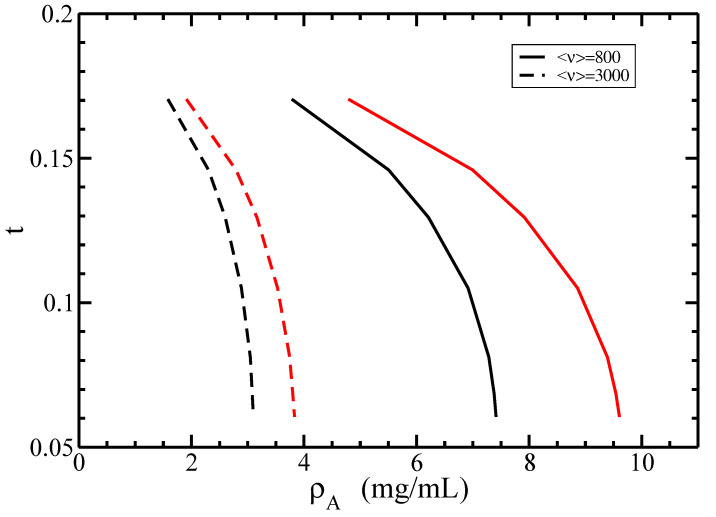
The isotropic–nematic phase diagram in terms of coexisting densities of actin $\rho_{A}$ for different values of the twisting parameter *t*. The average lengths of filaments are <ν> = 800 (solid lines) and 3000 (dashed lines); the persistence length P=18 μm, and the normalized standard deviation σ=0.5. The coexisting isotropic and nematic densities are plotted in black and red colors, respectively.

For actin filaments, the parameter *t* decreases with the increase of the concentration $c_{e}$. We also rescale the I–N phase diagram in terms of coexisting densities of actin $\rho_{A}$ for different values of the twisting parameter *t* in Figure 14.

It is seen that both isotropic and nematic coexisting densities $\rho_{A}$ decrease with increasing twisting parameter *t*, whereas, for a fixed value *t*, the increase of the average size <ν> decreases both coexisting densities $\rho_{A}$. Indeed, according to Figure 10 and Figure 13, the increase of the twisting parameter *t* decreases the electrolyte concentration $c_{e}$ and, correspondingly, increases the effective diameter $D_{eff}$. Furthermore, our results plotted in Figure 14 show that stabilizing effects on the nematic state of larger effective diameters $D_{eff}$ dominate destabilizing twisting effects with larger twisting parameters *t* [11].

## 4. Discussion

In the present article, we developed a classical density functional theory to calculate the isotropic–nematic phase diagram for actin filaments in physiological electrolyte solutions. The approach is based on an extension of Onsager’s second-order theory for monodisperse, charged rigid rods [10]. The key ingredients in this work are a unique definition of the orientational free energy $f_{or}$ and the $n_{\nu }$ size and angular $\eta_{\nu }\left(\theta \right)$ distribution functions, which properly account for the polydispersity and semiflexibility of the actin filaments. Unlike many studies on polydisperse rods where the size distribution function $n_{\nu }$ is an input parameter [17,19], actin filaments self-aggregate to produce a size distribution function $n_{\nu }$ that satisfies the equilibrium condition given by Equation (27). Our results reveal that $n_{\nu }$ has the form of Schulz distribution function, which is in accordance with experimental work performed on actin filaments [25,26]. To model an angular distribution function in the nematic phase $\eta_{\nu }\left(\theta \right)$, we generalized the trial function introduced in [17] for polydisperse filaments, which depends on two parameters α and γ. Additionally, we accounted for the filament semiflexibility by extending the formula for orientational free energy $f_{or}$ introduced in [13] to the case of a polydisperse system. Finally, we calculated the isotropic–nematic phase diagrams for several values of the normalized standard deviation of Schulz distribution σ, the persistence lengths of actin filaments *P*, and the concentrations of monovalent ions $c_{e}$. We compared the obtained results with the corresponding experimental data presented in [26]. We found that the set of parameters P=18 μm and $c_{e}=0.1\;$M gives the best match against the experiment.

In principle, our method can be applied with suitable modifications to other associating particles or exchange colloids that form charged rod-shaped aggregates such as DNA and ionic micelles. In the case of microtubules, the theory should be modified to account for the impact of the lumen on the their electrostatic interactions. It can be also extended to study phase diagrams in confined spaces, as well as under other electrolyte and filament conditions.

For instance, the orientational trial function used in this article is valid for weak polydispersity, i.e., when the normalized standard deviation σ is small. However, the solution can also be obtained for highly polydisperse systems, while the computational burden dramatically increases.

Additionally, in our study, we followed the conditions of the experiment, where the average length of actin filaments <ν> was fixed both in the isotropic and nematic phases [26]. As a result, the last two terms in the expression for free energy (43) did not contribute to the phase equilibrium. In principle, we can also apply our theory for polydisperse systems having different average lengths in the coexisting isotropic and nematic phases. In this case, the last two terms in the equation for free energy (43) cannot be disregarded. Additionally, the approximation introduced in this work for the excess standard chemical potential $\Delta \mu_{\nu }^{\left(0\right)}$ in Equation (39) should be properly changed/modified if the length distribution of the polydisperse system does not follow Schulz distribution function $n_{\nu }$.

On the other hand, we used Brenner–Parsegian’s formula [27] to approximate the electrostatic part of inter-filament potential in Equation (15). This approximation accounts for water solvent as a dielectric medium characterized by a constant dielectric permittivity. In principle, a more accurate, distance-dependent dielectric medium can be used to capture the high water polarization near the filament surface [32].

Furthermore, actin filaments may form bundles and networks due to the attraction forces between filaments produced by linker proteins or divalent ions in eukaryotic cells. In our method, we accounted only for repulsive interaction between filaments, i.e., the hard core plus electrostatic repulsion in Equation (14). The attraction between filaments can be included, for example, using square-well-type potentials [2,33].

Our approach is also able to describe charged filaments in pathological conditions. For instance, pH changes and G-actin mutations were accounted for from molecular structure models for actin filaments [34].

Overall, more realistic models can be developed for filament–filament and filament–binding protein interactions in constrained spaces (encapsidated polyelectrolytes) [35,36]. For instance, a variety of geometries such as spherical and cylindrical capsids can be used to model different cellular compartments such as dendrites (spines and filopodia), soma, axons, and terminals.

## Data Availability

Some or all data, models, or code that support the findings of this study are available from the corresponding author upon reasonable request.

## Appendix A. Cluster Integral B_*ν*1*ν*2_(*ω*_1_,*ω*_2_) in Equation

The volume integration in Equation (12) can be written as follows:

(A1) $$\begin{array}{c} \int d{\vec{r}}_{12}\to \int_{0}^{L_{1}}dz\int_{0}^{L_{2}\sin\gamma_{12}}dy\int_{-\infty }^{+\infty }dx \end{array},$$

where the *x* axis is chosen along the direction of the closest distance between rods, the *z* axis along the direction of first rod, and the *y* axis in the perpendicular direction to the z−x plane. $L_{1}$, $L_{2}$ are the lengths of two rods and $\gamma_{12}$ the angle between the axis of two rods. Substitution of Equation (A1) into Equation (12) yields

(A2) $$\begin{array}{c} B\left(\omega_{1},\omega_{2}\right)=\int_{0}^{L_{1}}dz\int_{0}^{L_{2}\sin\gamma_{12}}dy\int_{-\infty }^{+\infty }dx\left[1-e^{-\beta w_{12}\left(x,\omega_{1},\omega_{2}\right)}\right]. \end{array}$$

Integration in Equation (A2) on the y−z plane leads to

(A3) $$\begin{array}{c} B\left(\omega_{1},\omega_{2}\right)=L_{1}L_{2}\sin\gamma_{12}2\int_{0}^{+\infty }dx\left[1-e^{-\beta w_{12}\left(x,\omega_{1},\omega_{2}\right)}\right]. \end{array}$$

Substitution of Equation (14) into Equation (A3) yields

(A4) $$\begin{array}{c} B\left(\omega_{1},\omega_{2}\right)=2L_{1}L_{2}\sin\gamma_{12}\left(D+\int_{D}^{+\infty }dx\left[1-e^{-\Gamma_{⊥}\left(x,\gamma_{12}\right)}\right]\right). \end{array}$$

Substitution of Equation (15) into the integral in the r.h.s. of Equation (A4) yields the following expression:

(A5) $$\begin{array}{c} J=\int_{D}^{+\infty }dx\left[1-e^{-C_{1}e^{-kx}}\right] \end{array},$$

where

(A6) $$C_{1}=\frac{\Gamma_{⊥}}{\sin\gamma_{12}}e^{k_{D}D}.$$

The analytic solution of Equation (A5) is given by

(A7) $$J=\frac{1}{k_{D}}\left\{\ln\left(C_{1}e^{-k_{D}D}\right)+\gamma_{E}+E_{1}\left(C_{1}e^{-k_{D}D}\right)\right\},$$

where $\gamma_{E}$ is Euler’s constant:

(A8) $$\gamma_{E}=-\int_{0}^{+\infty }e^{-t}\ln tdt=0.5772\ldots ,$$

and $E_{1}\left(x\right)$ is the exponential integral:

(A9) $$E_{1}\left(x\right)=\int_{x}^{+\infty }\frac{e^{-t}}{t}dt.$$

Next, we substitute Equations (A5)–(A7) into Equation (A4) to obtain

(A10) $$B\left(\omega_{1},\omega_{2}\right)=2L_{1}L_{2}\sin\gamma_{12}\;\left(D+\frac{1}{k_{D}}\left\{\ln\left(\frac{\Gamma_{⊥}}{\sin\gamma_{12}}\right)+\gamma_{E}+E_{1}\left(\frac{\Gamma_{⊥}}{\sin\gamma_{12}}\right)\right\}\right).$$

We introduce the effective diameter $D_{eff}$ to rewrite Equation (A10) as follows:

(A11) $$B\left(\omega_{1},\omega_{2}\right)=2L_{1}L_{2}\sin\gamma_{12}D_{eff}\;\left(1+\frac{1}{k_{D}D_{eff}}\left\{-\ln\sin\gamma_{12}-\ln2+\frac{1}{2}+E_{1}\left(\frac{\Gamma_{⊥}}{\sin\gamma_{12}}\right)\right\}\right),$$

where

(A12) $$D_{eff}=D+\frac{1}{k_{D}}\left\{\ln\left(\Gamma_{⊥}\right)+\gamma_{E}+\ln2-\frac{1}{2}\right\}.$$

Finally, Equation (A11) can be written as

(A13) $$\begin{array}{c} B_{\nu_{1}\nu_{2}}\left(\omega_{1},\omega_{2}\right)=2D_{eff}l_{m}^{2}\nu_{1}\nu_{2}\;\;B*\left(\sin\gamma_{12}\right), \end{array}$$

where

(A14) $$\begin{array}{c} B*\left(\sin\gamma_{12}\right)=\sin\gamma_{12}\;\left\{1+\frac{1}{k_{D}D_{eff}}\left[-\log\left(\sin\gamma_{12}\right)-\ln2+\frac{1}{2}+E_{1}\left(\frac{\Gamma_{⊥}}{\sin\gamma_{12}}\right)\right]\right\}. \end{array}$$

## Appendix B. Schulz Distribution Function in Equation

Here, we provide some properties of Schulz distribution function $n_{\nu }$. We define the average size in the *k*-th power $<\nu^{k}>$ as follows:

(A15) $$\begin{array}{c} <\nu^{k}>=\int_{0}^{\infty }d\nu \;\nu^{k}\;n_{\nu }. \end{array}$$

Substitution of Equation (40) into Equation (A15) gives

(A16) $$\begin{array}{c} <\nu^{k}>=\frac{\Gamma (z+k+1)}{\Gamma (z+1)}\;\frac{1}{y^{k}}, \end{array}$$

where Γ is the Gamma function:

(A17) $$\begin{array}{c} \Gamma \left(z+1\right)=\int_{0}^{\infty }dx\;x^{z}\;e^{-x}. \end{array}$$

Equation (A16) yields the following expressions for k=0, 1, 2:

(A18) $$\begin{array}{c} <\nu^{0}>=<1>=1, \end{array}$$

(A19) $$\begin{array}{c} <\nu >=\frac{\left(z+1\right)}{y}, \end{array}$$

(A20) $$\begin{array}{c} <\nu^{2}>=\frac{\left(z+1)(z+2\right)}{y^{2}}. \end{array}$$

Substitution of Equations (A19) and (A20) in Equation (5) generates the following relation between the normalized standard deviation σ and parameter *z*:

(A21) $$\begin{array}{c} \sigma =\frac{1}{\sqrt{z+1}}. \end{array}$$

## Appendix C. Orientational Energy *f_or_* in Equation

The orientational free energy $f_{or}$ in the nematic phase for monodisperse systems can be expressed as [13]:

(A22) $$f_{or}=\left\{\begin{array}{cc} \;\int \eta \ln\eta \;d\omega ,\;\;\;\;\;\;\;\;\;\;\;\; & L\ll P\\ \frac{L}{8P}\int \frac{1}{\eta }{\left(\frac{\partial \eta }{\partial \theta }\right)}^{2}\;d\omega ,\;\;\;\;\;\;\;\;\;\;\;\; & L\gg P \end{array}\right.$$

where *L* is the filament length and *P* the persistence length. Khokhlov and Semenov introduced the correction terms to the r.h.s. of Equation (A22) to make this expression even more accurate: [13,14,15]

(A23) $$f_{or}=\left\{\begin{array}{cc} \;\int \eta \ln\eta \;d\omega +\frac{L}{12P}\int \frac{1}{\eta }{\left(\frac{\partial \eta }{\partial \theta }\right)}^{2}\;d\omega ,\;\;\;\;\;\;\;\;\;\;\;\; & L\ll P\\ \frac{L}{8P}\int \frac{1}{\eta }{\left(\frac{\partial \eta }{\partial \theta }\right)}^{2}\;d\omega -2\ln\left[\int \eta^{\frac{1}{2}}d\omega \right]+\ln\left[4\pi \right].\;\;\;\;\;\;\;\;\;\;\;\; & L\gg P \end{array}\right.$$

We generalize Equation (A23) to the case of polydisperse system in the following form:

(A24) $$f_{or}=\left\{\begin{array}{cc} \;\sum_{\nu \geq 1}n_{\nu }\int \eta_{\nu }\ln\eta_{\nu }\;d\omega +\sum_{\nu \geq 1}n_{\nu }\frac{L}{12P}\int \frac{1}{\eta_{\nu }}{\left(\frac{\partial \eta_{\nu }}{\partial \theta }\right)}^{2}\;d\omega ,\;\;\;\;\;\;\;\;\;\;\;\; & <L>\ll P\\ \sum_{\nu \geq 1}n_{\nu }\frac{L}{8P}\int \frac{1}{\eta_{\nu }}{\left(\frac{\partial \eta_{\nu }}{\partial \theta }\right)}^{2}\;d\omega -2\sum_{\nu \geq 1}n_{\nu }\ln\left[\int \eta_{\nu }^{\frac{1}{2}}d\omega \right]+\ln\left[4\pi \right],\;\;\;\;\;\;\;\;\;\;\;\; & <L>\gg P \end{array}\right.$$

where $<L>=l_{m}<\nu >$. Substitution of Equation (41) into Equation (A24) and the use of Equations (45) and (47) lead to

(A25) $$f_{or}=\left\{\begin{array}{cc} \ln\alpha -1-\frac{\gamma^{2}}{2}\sigma^{2}+N_{p}\frac{\alpha }{6},\;\;\;\;\;\;\;\;\;\;\;\; & <L>\ll P\\ N_{p}\frac{\alpha }{4}+\ln\alpha -\frac{\gamma^{2}}{2}\sigma^{2}-2\ln2,\;\;\;\;\;\;\;\;\;\;\;\; & <L>\gg P \end{array}\right.$$

where $N_{p}$ is given by Equation (48). We interpolate these two asymptotic values in Equation (A25) to obtain

(A26) $$f_{or}=\ln\alpha -\frac{\gamma^{2}}{2}\sigma^{2}+N_{p}\frac{\alpha }{6}+\frac{5}{12}\ln\left[\cosh\left(N_{p}\frac{\alpha }{5}\right)\right]-\frac{19}{12}\ln2.$$

In the monodisperse case, i.e., for σ=0, Equations (48), (A25), and (A26) reduce to those provided in [13]. It was also suggested in [13] to substitute $\left(\alpha -1\right)N_{p}$ instead of $\alpha N_{p}$ in the monodisperse version of Equation (A26) to have reliable results for small α, even when $N_{p}∼1$.

## Appendix D. Interaction Energy *f_int_* in Equations

We substitute Equations (18) and (19) into Equation (50) to have

(A27) $$\begin{array}{c} f_{int}=\rho \;\frac{\pi }{4}D_{eff}l_{m}^{2}\;\frac{4}{\pi }\int \sum_{\nu_{1}\geq 1}n_{\nu_{1}}\nu_{1}\eta_{\nu_{1}}\left(\theta_{1}\right)\sum_{\nu_{2}\geq 1}n_{\nu_{2}}\nu_{2}\eta_{\nu_{2}}\left(\theta_{2}\right)B*\left(\sin\gamma_{12}\right)d\omega_{1}d\omega_{2}. \end{array}$$

Equation (A27) can be expressed as

(A28) $$\begin{array}{c} f_{int}=\rho \;\frac{\pi }{4}D_{eff}l_{m}^{2}<\nu {>}^{2}\;\frac{4}{\pi }\int g_{z}\left(\theta_{1}\right)g_{z}\left(\theta_{2}\right)B*\left(\sin\gamma_{12}\right)d\omega_{1}d\omega_{2}, \end{array}$$

where

(A29) $$\begin{array}{c} g_{z}\left(\theta \right)=\frac{1}{<\nu >}\sum_{\nu \geq 1}n_{\nu }\nu \eta_{\nu }\left(\theta \right). \end{array}$$

Additionally, we substitute Equation (40) for the distribution functions $n_{\nu }$ and Equations (41) and (42) for $\eta_{\nu }\left(\theta \right)$ into Equation (A29). This results in

(A30) $$\begin{array}{c} g_{z}\left(\theta \right)=\frac{1}{<\nu >}\sum_{\nu \geq 1}\frac{y^{z+1}}{\Gamma (z+1)}\nu^{z}e^{-y\nu }\;\nu \;\frac{\alpha }{4\pi }\left(\frac{\gamma \nu }{<\nu >}+\left(1-\gamma \right)\right)e^{-\frac{\alpha }{2}\left(\frac{\gamma \nu }{<\nu >}+\left(1-\gamma \right)\right)\theta^{2}}. \end{array}$$

In Equation (A30), the summation can be written in the form of integration as follows:

(A31) $$\begin{array}{c} g_{z}\left(\theta \right)=\frac{y^{z+1}}{\Gamma (z+1)}\frac{1}{4\pi }\frac{\alpha }{<\nu {>}^{2}}e^{-\frac{\alpha }{2}\left(1-\gamma \right)\theta^{2}}\int_{0}^{\infty }d\nu \;\left(\gamma \nu^{z+2}+\left(1-\gamma \right)\nu^{z+1}<\nu >\right)e^{-(y+\frac{\alpha }{2}\frac{\gamma }{<\nu >}\theta^{2})\nu }. \end{array}$$

Using the definition of the Gamma function given by Equation (A17), Equation (A31) becomes

(A32) $$\begin{array}{c} g_{z}\left(\theta \right)=\frac{\alpha }{4\pi }\frac{y^{z+1}}{<\nu {>}^{2}}e^{-\frac{\alpha }{2}\left(1-\gamma \right)\theta^{2}}\left(\frac{\Gamma (z+3)}{\Gamma (z+1)}\frac{\gamma }{{\left(y+\frac{\gamma \alpha \theta^{2}}{2<\nu >}\right)}^{z+3}}+\frac{\Gamma (z+2)}{\Gamma (z+1)}\frac{(1-\gamma )<\nu >}{{\left(y+\frac{\gamma \alpha \theta^{2}}{2<\nu >}\right)}^{z+2}}\right) \end{array}$$

which can be rewritten as

(A33) $$\begin{array}{c} g_{z}\left(\theta \right)=\frac{\alpha }{4\pi }\frac{e^{-\frac{\alpha }{2}\left(1-\gamma \right)\theta^{2}}}{<\nu {>}^{2}}\left(\frac{\gamma (z+1)(z+2)}{y^{2}{\left(1+\frac{\gamma \alpha \theta^{2}}{2<\nu >y}\right)}^{z+3}}+\frac{\left(z+1)<\nu >(1-\gamma \right)}{y{\left(1+\frac{\gamma \alpha \theta^{2}}{2<\nu >y}\right)}^{z+2}}\right). \end{array}$$

Substitution of the function *y* in Equation (A19) into Equation (A33) leads to

(A34) $$\begin{array}{c} g_{z}\left(\theta \right)=\frac{\alpha }{4\pi }e^{-\frac{\alpha }{2}\left(1-\gamma \right)\theta^{2}}\left(\frac{\gamma \frac{\left(z+2\right)}{\left(z+1\right)}}{{\left(1+\frac{\gamma \alpha \theta^{2}}{2(z+1)}\right)}^{z+3}}+\frac{\left(1-\gamma \right)}{{\left(1+\frac{\gamma \alpha \theta^{2}}{2(z+1)}\right)}^{z+2}}\right). \end{array}$$

Finally, we transform the angular integrals in Equation (A28). To this end, we use the following formula:

(A35) $$\begin{array}{c} \int d\omega_{1}d\omega_{2}\ldots =8\pi \;\int_{0}^{\pi /2}\sin\theta_{1}d\theta_{1}\int_{0}^{\pi /2}\sin\theta_{2}d\theta_{2}\int_{0}^{2\pi }d\varphi_{12}\ldots \end{array}$$

Substitution of Equation (A35) into Equation (A28) yields

(A36) $$\begin{array}{c} f_{int}=\rho \;\frac{\pi }{4}Dl_{m}^{2}<\nu {>}^{2}\;32\;\int_{0}^{\pi /2}\sin\theta_{1}d\theta_{1}\int_{0}^{\pi /2}\sin\theta_{2}d\theta_{2}g_{z}\left(\theta_{1}\right)g_{z}\left(\theta_{2}\right)K\left(\theta_{1},\theta_{2}\right), \end{array}$$

where

(A37) $$\begin{array}{c} K\left(\theta_{1},\theta_{2}\right)=\int_{0}^{2\pi }B*\left(\sin\gamma_{12}\right)d\varphi_{12} \end{array}$$

and

(A38) $$\begin{array}{c} \sin\gamma_{12}=\sqrt{1-{\left(\cos\theta_{1}\cos\theta_{2}+\sin\theta_{1}\sin\theta_{2}\cos\varphi_{12}\right)}^{2}}. \end{array}$$

We note that the function $K(\theta_{1},\theta_{2})$ is independent of the filaments’ concentration ρ and the average filaments’ length <ν>. Thus, it can be numerically precalculated and used in subsequent calculations. In practice, we calculated *K* and stored the values in a matrix of size [200×200].

## Appendix E. Asymptotic Expression for *f_mix_* in the Monodisperse Limit

We substitute the distribution function $n_{\nu }$, given by Equation (40) into Equation (58). We obtain

(A39) $$\begin{array}{c} f_{mix}=\int d\nu \;n_{\nu }\ln\left(\frac{y^{z+1}}{\Gamma (z+1)}\;\nu^{z}\;e^{-y\nu }\right). \end{array}$$

Substitution of Equations (A18), (40), and (A19) into Equation (A39) yields

(A40) $$\begin{array}{c} f_{mix}=\ln\left[\frac{y^{z+1}}{\Gamma (z+1)}\right]+z\;\frac{y^{z+1}}{\Gamma (z+1)}\;J-\left(z+1\right), \end{array}$$

where

(A41) $$\begin{array}{c} J=\int d\nu \;\nu^{z}\;e^{-y\nu }\ln\nu . \end{array}$$

We obtain the analytic solution for *J* using the expression number 4.352 from the integral table provided in [37]. We have

(A42) $$\begin{array}{c} J=\frac{\Gamma (z+1)}{y^{z+1}}\;\left(H_{z}-\gamma_{E}-\ln y\right), \end{array}$$

where $\gamma_{E}=0.5772$ is Euler’s constant, Γ(z+1)=z! the gamma function, and $H_{z}=1+\frac{1}{2}+\frac{1}{3}+\ldots +\frac{1}{z}$ the *z*-th harmonic number. Substitution of Equation (A42) into the second term in the r.h.s. of Equation (A40) gives

(A43) $$\begin{array}{c} f_{mix}=\ln\left[\frac{y}{\Gamma \left(z+1\right)e^{z+1}}\right]+z\;\left(H_{z}-\gamma_{E}\right). \end{array}$$

We use the following asymptotic formula for the harmonic number:

(A44) $$\begin{array}{ccc} H_{z}\to \ln z+\gamma_{E}+\frac{1}{2z}\;\;\;\;\;\;\;\; & & z\to \infty \end{array}$$

to calculate the free energy $f_{mix}$ in the monodisperse limit. We substitute Equation (A44) into Equation (A43), and we use Equations (A19) and (A21), and Stirling’s formula $\Gamma \left(z+1\right)\to \sqrt{2\pi z}{\left(z/e\right)}^{z}$ to finally obtain

(A45) $$\begin{array}{c} f_{mix}\to -\ln\left[\sqrt{\frac{2\pi ez}{z+1}}\;<\nu >\sigma \right]\;\;\;\;\;\;\;\;\;\;\;\left(z\to \infty \right). \end{array}$$
